# Role of N-linked glycosylation sites in human ACE2 in SARS-CoV-2 and hCoV-NL63 infection

**DOI:** 10.1128/jvi.02202-24

**Published:** 2025-03-28

**Authors:** Sabrina Noettger, Fabian Zech, Rayhane Nchioua, Chiara Pastorio, Christoph Jung, Timo Jacob, Steffen Stenger, Frank Kirchhoff

**Affiliations:** 1 Institute of Molecular Virology, Ulm University Medical Center 27197, Ulm, Germany; 2 Institute of Electrochemistry, Ulm University 685576, Ulm, Germany; 3 Electrochemical Energy Storage, Helmholtz-Institute-Ulm 557388 , Ulm, Germany; 4 Karlsruhe Institute of Technology 150232, Karlsruhe, Germany; 5 Institute of Medical Microbiology and Hygiene, Ulm University Medical Center 27197, Ulm, Germany; The Ohio State University, Columbus, Ohio, USA

**Keywords:** SARS-CoVs, hCoV-NL63, ACE2, glycosylation, infection, cell-to-cell fusion

## Abstract

Angiotensin-converting enzyme 2 (ACE2) is a transmembrane protein known for its physiological role in the renin-angiotensin system that also serves as a receptor for entry of SARS-CoV-1, SARS-CoV-2, and the seasonal human coronavirus NL63 (hCoV-NL63). ACE2 contains seven N-linked glycosylation sites. Molecular simulation and binding analyses suggest that some of them are involved in the interaction with the Spike (S) proteins of hCoVs, but their relevance in S-mediated fusion and viral entry is poorly investigated. To address this, we determined the impact of all seven N-linked glycosylation sites in ACE2 on S-mediated SARS-CoV-2 and hCoV-NL63 infection as well as cell-to-cell fusion. We found that all mutant ACE2 proteins are expressed and localized at the cell surface, albeit ACE2 lacks all glycans at decreased levels. On average, changes in T92I, N322A, and N690A, as well as combined mutation of all N-linked glycosylation sites increased endocytic VSVpp infection mediated by early HU-1 as well as Omicron BA.2, BA.5, and XBB.1.5 SARS-CoV-2 S proteins. In comparison, only the lack of glycan at N322 in ACE2 enhanced syncytia formation and only in the case of HU-1 and XBB.1.5 S proteins. Changes in N90A, T92I, and N322A increased infection by the early SARS-CoV-2 HU-1 strain about twofold to threefold but had lesser effects on infection by genuine Omicron variants. Despite reduced cell surface expression of ACE2, elimination of all N-linked glycosylation sites usually enhanced SARS-CoV-2 infection via the endocytic pathway while having little effect on entry at the cell surface in the presence of TMPRSS2. Our results provide insights into the role of N-linked glycans in the ability of human ACE2 (hACE2) to serve as receptors for coronavirus infection.

**IMPORTANCE:**

Several human coronaviruses use angiotensin-converting enzyme 2 (ACE2) as a primary receptor for infection of human cells. ACE2 is glycosylated at seven distinct positions, and the role of glycans for the entry of SARS-CoV-2 and hCoV-NL63 into their target cells is incompletely understood. Here, we examined the impact of individual and combined mutations in hACE2 glycosylation sites on Spike-mediated VSV-pseudoparticle and genuine SARS-CoV-2 and hCoV-NL63 infection and cell-to-cell fusion. Our results provide new information on the role of glycans in hACE2 for infection by highly pathogenic and seasonal coronaviruses.

## INTRODUCTION

Angiotensin-converting enzyme 2 (ACE2) is a zinc-containing metalloprotease catalyzing the conversion of angiotensin II to angiotensin 1–7 (1). Thus, hACE2 is a negative regulator of the renin-angiotensin-aldosterone system that plays an important role in regulating blood pressure and electrolyte balance (1–3). hACE2 is expressed in numerous tissues, including the heart, lungs, kidneys, as well as the gastrointestinal tract, and regulates various physiological functions beyond mediating anti-inflammatory and cardioprotective effects (4–6).

ACE2 is not only a promising target for the treatment of cardiovascular diseases (7) but also the major entry receptor for three of the seven human coronaviruses, that is, SARS-CoV-1, SARS-CoV-2, and hCoV-NL63 (8–10). hACE2 is a type I integral membrane protein of 805 amino acids (11). It comprises a single-pass transmembrane domain that anchors the protein in the cell membrane. hACE2 contains a large extracellular peptidase domain, as well as a collectrin-like domain (12). The structure of the hACE2 receptor in complex with the SARS-CoV-2 S protein has been solved and revealed the major interaction regions of the receptor-binding domain (RBD) (13). The S protein is initially synthesized as a single-chain precursor, trimerizes, and is cleaved by furin in the virus-producer cells (14). Thus, mature virions contain two subunits: the S1 subunit that binds hACE2 and is non-covalently bound to the S2 subunit anchored in the viral membrane. For efficient hACE2 interaction, the RBD of at least one S1 subunit has to undergo conformational changes from the prefusion down conformation to the up conformation (14). After binding of the viral S protein to the peptidase domain of hACE2, it is further cleaved at the S2′ site by the host serine protease transmembrane serine protease 2 (TMPRSS2) (8, 15, 16). Alternatively, the S2 subunit can also be processed and activated by endosomal cathepsin proteases (17–19). Following major conformational changes, the fusion peptide (FP) is inserted into the cellular membrane. The three S2 subunits then form a six-helix bundle that brings the viral and cellular membranes together, creating a fusion pore that allows viral entry and replication.

ACE2 and the SARS-CoV-1 and SARS-CoV-2 S proteins are both heavily glycosylated, and some glycosylation sites are located near the interacting surfaces (13, 20–22). The extracellular domain of hACE2 contains seven N-linked glycosylation sites (N53, N90, N103, N322, N432, N546, and N690) (21). An early study reported that glycosylation at N90 interferes with SARS-CoV-1 binding and infectivity (23). Recent genetic and biochemical studies indicate that elimination of this glycosylation site may enhance susceptibility to SARS-CoV-2 infection (24, 25). Molecular modeling and dynamics simulations suggested that glycans N90 and N322 directly interact with the SARS-CoV-2 S protein (26, 27).

Previous studies have examined the impact of hACE2 glycosylation on the interaction with the S protein and neutralizing activity against SARS-CoV-2 (21, 25, 28–30). However, the role of the individual glycosylation sites in hACE2 in S-mediated cell-to-cell fusion and infection of SARS-CoV-2 and hCoV-NL63 is not fully understood. To address this, we mutated the seven N-linked glycosylation sites in hACE2 individually and in combination and examined the impact on S-mediated viral infection and cell-cell fusion. Our results show that changes in N-linked glycosylation sites affect S-mediated VSVpp infection more severely than infection by genuine SARS-CoV-2 variants. Lack of glycans at N90 and N322 enhanced infection of transfected HEK293T by an early SARS-CoV-2 strain up to threefold, while only N322A significantly enhanced infection by the more recent Omicron BA.5 strain. Despite reduced cell surface expression, the hACE2 variant lacking all N-linked glycosylation sites allowed enhanced cathepsin-dependent infection by SARS-CoV-2 S proteins compared to the parental hACE2, while hCoV-NL63 infection was reduced.

## RESULTS

### Localization and conservation of N-linked glycosylation sites in hACE2

**Fig 1:**
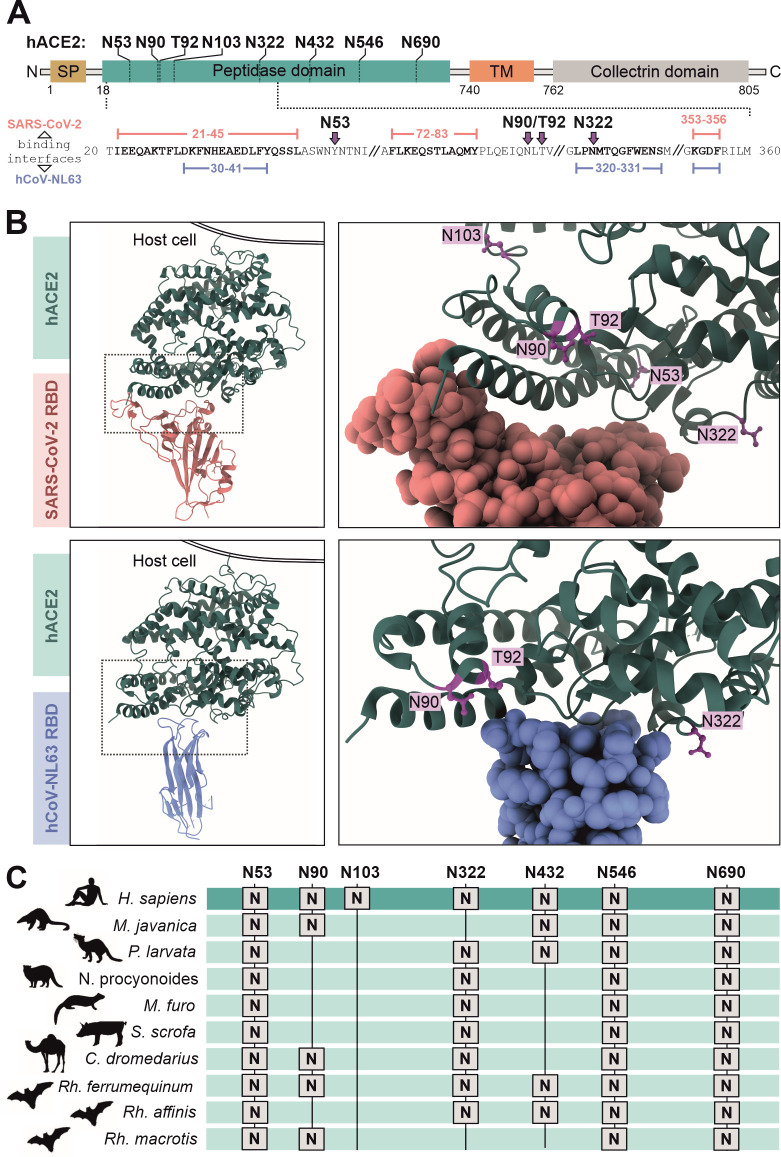
Localization and conservation of N-linked glycosylation sites in hACE2. (A) N-linked glycosylation sites and protein domains are indicated. Signal peptide (SP) domain is in gold, peptidase domain in turquoise, transmembrane (TM) domain in orange, and collectrin domain in gray. Binding interfaces with SARS-CoV-2 and hCoV-NL63 are indicated in rose and light blue, respectively. (B) 3D structural alignment of an hACE2 monomer with its N-linked glycosylation sites (purple) respectively. hACE2 monomers are shown in dark green. Left: front view; hACE2 complex is anchored to a cell membrane, right: detailed front view (PDB: 6M0J [SARS-CoV-2] and 3KBH [NL63]). (C) Conservation of hACE2 N-linked glycosylation sites in reservoir and intermediate hosts of SARS coronavirus 2 as well as humans. Accession numbers of hACE2 from various species are as follows: *Homo sapiens* (XP_005274628), *Manis javanica* (XP_017505752), *Paguma larvata* (AAX63775), *Nyctereutes procyonoides* (XP_055195065), *Mustela putorius furo* (XP_004758942), *Sus scrofa* (XP_020935033), *Camelus dromedarius* (XP_031301717), *Rhinolophus ferrumequinum* (XP_032963186), *Rhinolophus affinis* (QMQ39244), and *Rhinolophus macrotis* (ADN93471).

All seven N-linked glycosylation sites are located in the peptidase domain of hACE2 (Fig. 1A) that catalyzes the hydrolysis of angiotensin II to regulate blood pressure. The S proteins of SARS-CoV-2 and hCoV-NL63 each bind to three regions within hACE2, including two shared binding sites at residues aa30-41 and aa353-356 (Fig. 1A) (25, 31). The binding interface between SARS-CoV-2 and hACE2 is larger than that of hCoV-NL63 and hACE2, comprising 41 amino acids compared to 28 amino acids (Fig. 1A and B). Consequently, SARS-CoV-2 S interacts more strongly with hACE2 than hCoV-NL63 (32). Glycans N90 and N322 are located near the receptor-binding interface between hACE2 and the RBD of the Spike proteins (Fig. 1B). It has been reported that both of these glycans modulate the interaction of hACE2 with the SARS-CoV-2 S protein and may promote binding or restrict it due to steric hindrance (27, 28, 30, 33, 34). Notably, the N322 glycosylation site is lacking in hACE2 proteins of pangolins (*Manis javanica*) while masked palm civet (*Paguma larvata*) and raccoon dogs (*Nyctereutes procyonoides*) do not possess N90, representing possible intermediate hosts of SARS-CoV-2 (Fig. 1C) (35, 36). The hACE2 glycan at N546 was also reported to interact with the SARS-CoV-2 S protein, albeit with S glycans rather than amino acids (30, 33). Glycan N53 may also interact with the RBD of the S protein or indirectly stabilize the hACE2-Spike complex (27, 29, 31). Three out of seven N-linked glycosylation sites, N53, N546, and N690, are conserved among various coronavirus host species, including bats of the *Rhinolophus* genus as initial hosts (37, 38), raccoon dogs, ferrets, and pigs as intermediate hosts of human pathogenic coronaviruses, as well as humans themselves (Fig. 1C). Thus, glycans at these three positions might play a relevant role in hACE2 function.

### Expression and cellular localization of hACE2 glycosylation

**Fig 2:**
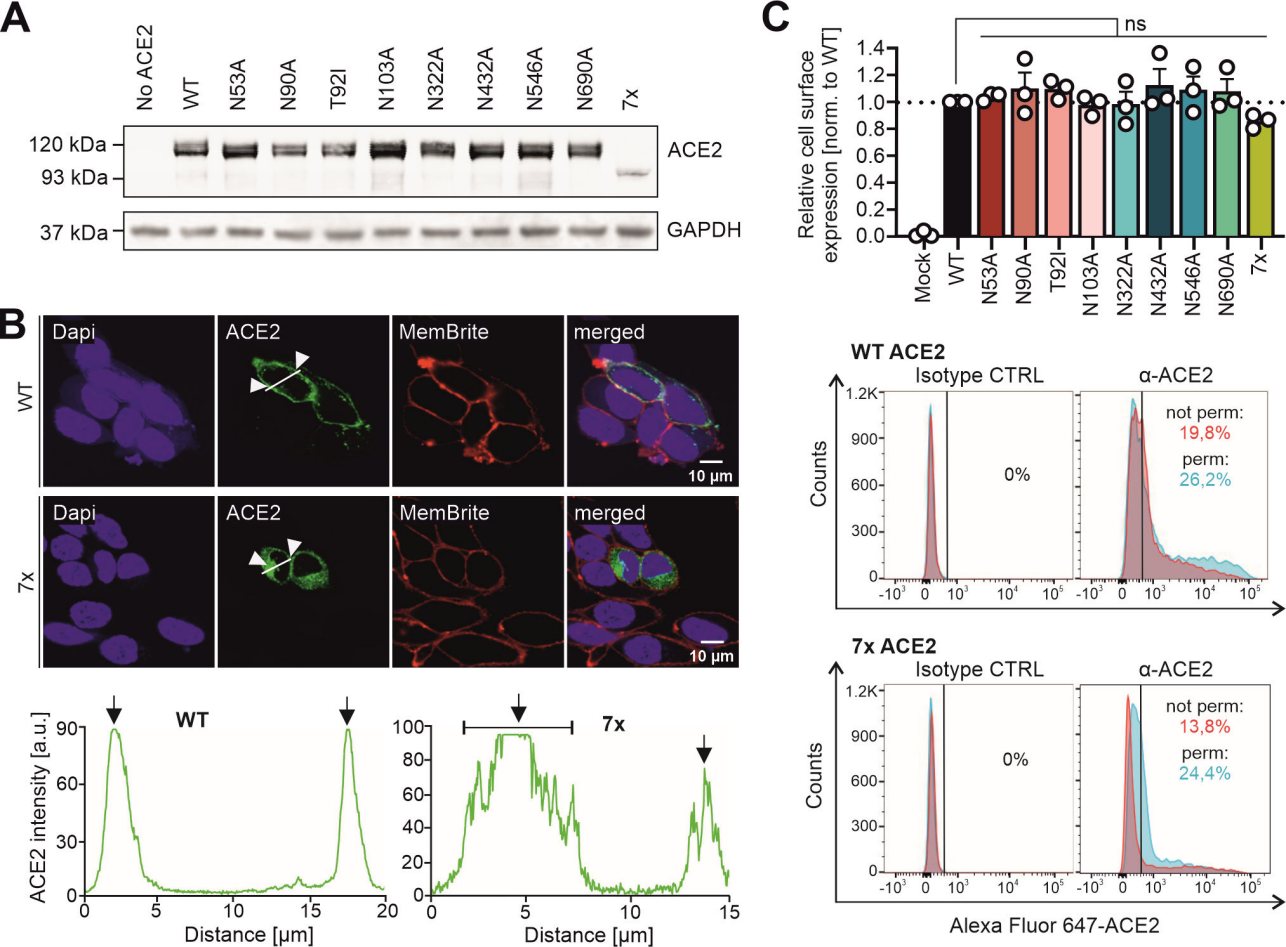
Expression and localization of WT and mutant hACE2 proteins. (A) Expression of hACE2 N-glycosylation constructs in HEK293T cells. Membranes were stained with anti-ACE2 and anti-GAPDH. Double bands refer to the glycosylated and partially unglycosylated isoforms of the protein. The multiple mutated hACE2 protein shows a decreased size of ~90 kDa and only one single band of non-glycosylated protein. (B) Immunofluorescence staining and intensity plots of HeLa cells expressing wild-type (WT) hACE2 and hACE2 lacking all seven glycosylation sites (7×). Cell nuclei are stained in blue (DAPI), the cell membrane in red (MemBrite), and hACE2 in green. Intensity plots show the intensity and localization of the hACE2 fluorescent signal throughout one cell, indicated by the white bar. The x-axis represents the distance (in µm) along the cell, and the y-axis denotes hACE2 intensity. The white arrows in the images and black arrows in the intensity blots indicate the position of the cellular membrane. (C) Quantification of cell surface expression of hACE2 N-glycosylation mutants in HeLa cells. Relative cell surface expression of each hACE2 construct has been calculated by taking the mean fluorescence intensity (MFI) ratio of non-permeabilized and permeabilized cells. Values displayed are normalized to WT ACE2. Statistical significance was tested by one-way ANOVA, ns = not significant. Histograms of flow cytometry analysis of surface and total hACE2 are shown for the WT and the non-glycosylated hACE2 condition. Percentages refer to hACE2-positive cells in permeabilized and non-permeabilized cells, respectively.

To determine their functional relevance for SARS-CoV-2 and hCoV-NL63 infection, we mutated the seven potential N-linked glycosylation sites both individually and in combination. Notably, it has been documented that all seven N-linked glycosylation sites of hACE2 expressed in HEK293T cells are highly occupied by glycans (39). In addition to changing all N residues to A, we also introduced a naturally occurring mutation of T92I (24) that abolishes the same glycosylation site as N90A. In agreement with published data (29), all N-glycosylation mutant hACE2 proteins were efficiently expressed in transfected HEK293T cells, albeit the combination mutant (7×) at reduced levels (Fig. 2A). The parental hACE2 protein and mutants thereof lacking individual glycosylation sites migrated as two distinct bands, most likely corresponding to forms differing in glycosylation (10). Predictably, mutation of all seven N-linked glycosylation sites resulted in faster migration as a single distinct band with a reduced molecular weight of ~90 kDa (28, 39). To determine whether lack of glycans affects the subcellular localization of hACE2, we performed confocal immune-fluorescence microscopic analyses in HeLa cells. hACE2 was almost exclusively detected at the cell surface, and none of the individual mutations had significant effects on localization or signal intensities (Fig. 2B; Fig. S1). As expected from previous data (28), lack of all seven N-linked glycosylation sites increased cytoplasmic localization of hACE2, although an unexpectedly high fraction was detectable at the cell surface (Fig. 2B). Flow cytometric analyses confirmed that all mutant hACE2 proteins were expressed at the cell surface, albeit the 7× hACE2 protein lacking all glycans at moderately reduced levels (Fig. 2C). Altogether, these results showed that only the lack of all seven glycosylation sites reduced hACE2 expression and exposure at the cell surface.

### Effect of N-linked glycosylation sites on S-mediated cell-cell fusion

**Fig 3:**
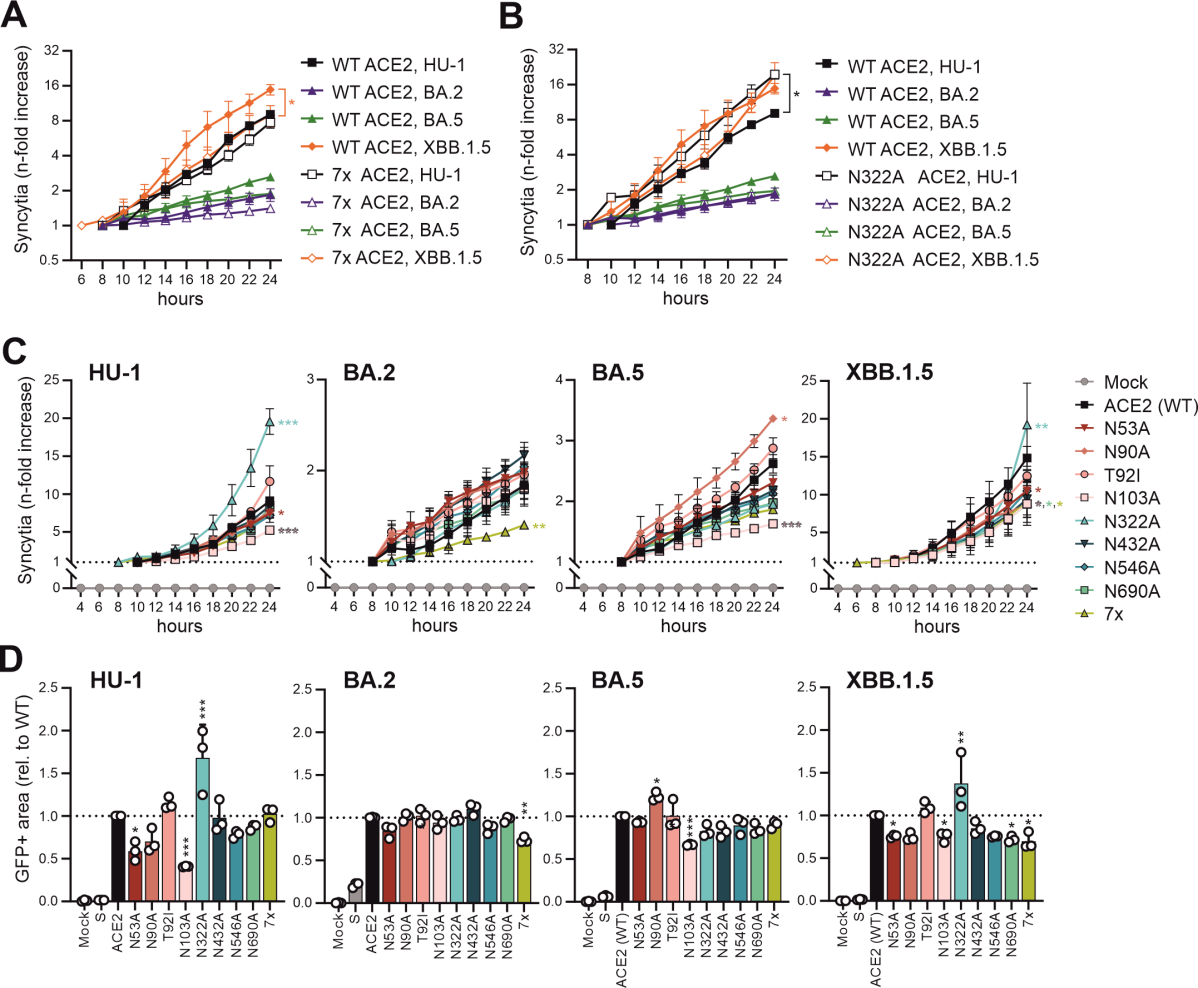
Impact of mutations in N-linked glycosylation sites of hACE2 on syncytia formation by SARS-CoV-2 S proteins. (A and B) Syncytia formation in HEK293T cells co-expressing WT or 7× (A)/WT or N322A (B) mutated hACE2 constructs and Spike proteins from indicated SARS-CoV-2 strains. Kinetic curves represent the mean of three independent experiments (± SEM). Statistical significance was tested by one-way ANOVA, *P < 0.05. (C) Syncytia formation in HEK293T cells co-expressing hACE2 mutant constructs and SARS-CoV-2 S proteins as indicated. Syncytia formation was monitored over a period of 24 h post-transfection. Kinetic curves represent the mean of three independent experiments (± SEM). The relative increase in size was calculated for every hACE2 mutant starting at the earliest time when GFP+ cells were detected in the microscope. Statistical significance was tested by one-way ANOVA, *P < 0.05; **P < 0.01; ***P < 0.001. (D) Fluorescence images were taken in an automated manner, and syncytia formation of hACE2 with indicated SARS-CoV-2 S protein was quantified by evaluating the GFP+ area using ImageJ software. Bars represent the mean of three independent experiments (± SEM). Statistical significance was tested by one-way ANOVA, *P < 0.05; **P < 0.01; ***P < 0.001.

Coronaviruses may spread by both cell-free virus infection, as well as S-mediated fusion of virally infected cells with uninfected bystander cells (40, 41). It has been suggested that mutations in the hACE2 glycosylation sites modulate cell-cell fusion through steric conformational changes or by affecting direct binding of S residues to hACE2 glycans (27). To evaluate the impact of hACE2 N-glycosylation mutants on cell fusogenicity, we measured cell-to-cell fusion of HEK293T cells expressing wild type (WT) or mutant hACE2 and various SARS-CoV-2 or hCoV-NL63 S proteins. The early HU-1 and the late Omicron XBB.1.5 S proteins were generally much more potent in inducing syncytia compared to those of BA.2 and BA.5 (Fig. 3A; Fig. S2). XBB.1.5 S also induced the largest syncytia, while BA.2 S was the least fusogenic (Fig. S2), which agrees with previous results (42–45). The HU-1 S used in the present study does not contain the D614G mutation that increases viral infectivity (46, 47). This most likely explains why it was not more fusogenic than XBB.1.5 S, as reported for an early S protein containing the D614G mutation (48). In contrast to SARS-CoV-2 S proteins, the hCoV-NL63 S did not mediate significant fusion of cells expressing WT or mutant hACE2 proteins (Fig. S3).

Lack of all glycans in hACE2 slightly reduced cell-to-cell fusion by all four SARS-CoV-2 S proteins (Fig. 3A), most likely due to reduced cell surface expression (28). In comparison, the lack of N322 moderately increased fusion by HU-1 S but had no or slightly attenuating effects on the induction of syncytia formation by the remaining SARS-CoV-2 S proteins (Fig. 3B). The relative impact of mutations in specific hACE2 glycans on fusion showed some variations between the different SARS-CoV-2 S proteins (Fig. 3C). For example, N53A reduced cell-to-cell fusion mediated by HU-1 and (to a lesser extent) XBB.1.5 S but had no significant effect on syncytia formation by BA.2 and BA.5 S (Fig. 3D). Unexpectedly, N103A in hACE2 that is only present in humans (Fig. 1C) and not in close proximity to the RBD also reduced fusion mediated by all S proteins, except that of BA.2. Altogether, S proteins of the four SARS-CoV-2 variants differed strongly in the ability to mediate cell-to-cell fusion, but mutations in N-linked glycosylation sites of hACE2 had only modest modulatory effects on this function.

### Impact of hACE2 glycans on S-mediated VSVpp infection

**Fig 4:**
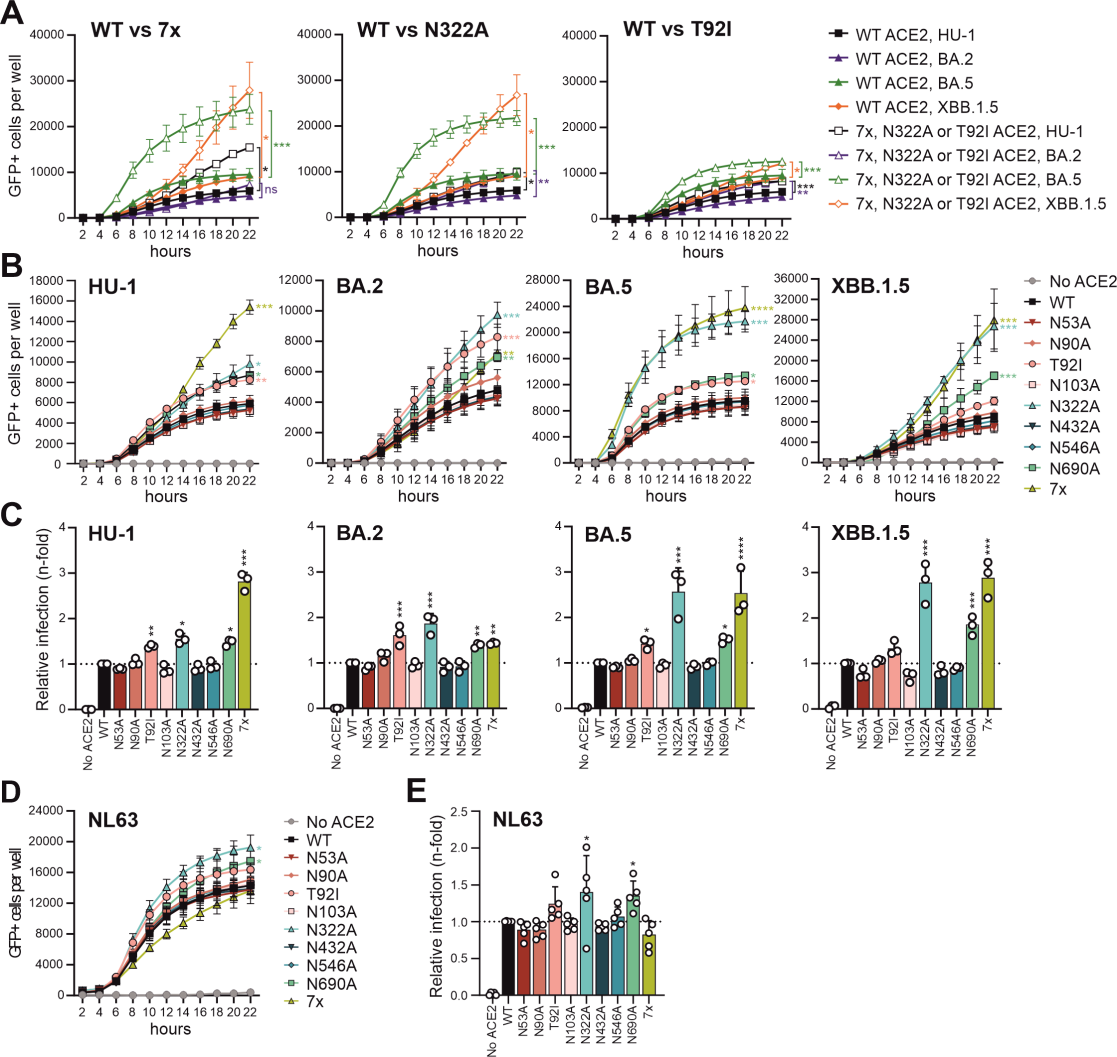
Impact of hACE2 N-glycosylation mutants on SARS-CoV-2 and hCoV-NL63 Spike-mediated infection in the absence of TMPRSS2. HEK293T cells were transfected with the indicated hACE2 constructs and infected with VSVpp carrying SARS-CoV-2 HU-1, BA.2, BA.5, XBB.1.5 Spike (A, B, C), or hCoV-NL63 Spike (D, E) proteins. (A) SARS-CoV-2-infected GFP-positive cells expressing WT, N322A, or T92I hACE2 were automatically quantified over a period of 22 h using the Cytation 3 plate reader microscope. Kinetic curves in panels A and B represent the mean of three independent experiments (± SEM). Statistical significance was tested by one-way ANOVA, *P < 0.05; **P < 0.01; ***P < 0.001; ****P < 0.0001. (B) HEK293T cells expressing various hACE2 mutant constructs were infected with SARS-CoV-2 S proteins as indicated. GFP-positive cells were automatically counted over a period of 22 h using the Cytation plate reader microscope. (C) Endpoint of SARS-CoV-2 S VSVpp infection after 22 h. Bars represent the mean of three independent experiments (± SEM). Statistical significance in panels C to E was tested by one-way ANOVA, *P < 0.05; **P < 0.01; ***P < 0.001; ****P < 0.0001. (D) Kinetic curves of VSVpp infection after 22 h. hCoV-NL63-infected BFP-positive cells were automatically quantified over a period of 22 h using the Cytation 3 plate reader microscope. Kinetic curves represent the mean of three independent experiments (± SEM). (E) Endpoint of hCoV-NL63 VSVpp infection after 22 h. Bars represent the mean of five independent experiments (± SEM).

Pseudotyping vesicular stomatitis virus (VSV), lacking its glycoprotein (ΔG), with S proteins allows for examination of key aspects of coronavirus entry under low biosafety conditions (8). To elucidate the impact of N-glycosylation of hACE2 on SARS-CoV-2 and hCoV-NL63 infection, we generated VSV pseudoparticles (pp) containing the SARS-CoV-2 HU-1, BA.2, BA.5, XBB.1.5, and hCoV-NL63 S proteins, respectively. The kinetics of S-mediated VSVpp infection in transfected HEK293T cells were quantified in an automated manner by counting GFP+ or BFP+ cells, as previously reported (42, 43). In contrast to their effects on cell-to-cell fusion (Fig. 3), the BA.2 and BA.5 S proteins mediated VSVpp infection with only moderately reduced (BA.2) or increased (BA.5) efficiency compared to HU-1 and XBB.1.5 S (Fig. 4A). Lack of all glycans in hACE2 strongly accelerated and increased BA.5 S-mediated VSVpp infection. Despite reduced expression at the cell surface, the 7× hACE2 also allowed increased VSVpp infection by XBB.1.5 S, although in this case with unchanged kinetics (Fig. 4A). Mutation of N322A had a very similar impact on S-mediated VSVpp infection as removal of all N-linked glycosylation sites, while T92I had lesser effects. These results indicate strong steric restriction of BA.5 and XBB.1.5 S-mediated infection by the N322 glycan in hACE2.

Unlike changes in T92I and N322A, mutations of N53A, N90A, N103A, N432A, and N546A in hACE2 had no significant effect on the kinetics (Fig. 4B) and efficiency (Fig. 4C) of VSVpp infection mediated by the four SARS-CoV-2 S proteins. In most cases, this is plausible since these glycans in hACE2 are not in close proximity or interacting with the RBD of the viral S protein (27, 28, 30, 33). However, the lack of an effect of N90A came as a surprise since it affects the same glycosylation site as T92I, and previous studies indicated that abrogation of N90 glycosylation enhances binding to the RBD of hACE2 (25, 29, 30). In addition, N690A significantly increased VSVpp infection by all four SARS-CoV-2 S proteins investigated (Fig. 4C). Thus, changes in T92I and N690A might impact the entry receptor function of hACE2 independently of direct effects on glycan interactions with the SARS-CoV-2 S protein.

In contrast to the effect on SARS-CoV-2 S, the lack of all glycans in hACE2 moderately reduced NL63 S-mediated VSVpp infection (Fig. 4D and E). This was most likely due to reduced surface expression of deglycosylated hACE2 since lack of glycans N322 and N690 slightly enhanced NL63 S-mediated infection. Correlation analyses revealed that the changes in N-linked glycosylation sites had similar effects on entry by the four different SARS-CoV-2 S proteins (Fig. S4). Especially the correlation between BA.5 and XBB.1.5 S protein was strikingly high (R^2^ = 0.9822) given that both differ by about 19 amino acid changes (49). In comparison, the effects of glycan changes on SARS-CoV-2 and hCoV-NL63 S-mediated VSVpp infection did not correlate, largely because the lack of all glycans had opposite effects. Altogether, the results show that glycan N322 plays the dominant role in hindering SARS-CoV-2 BA.5 and XBB.1.5 S-mediated infection.

**Fig 5:**
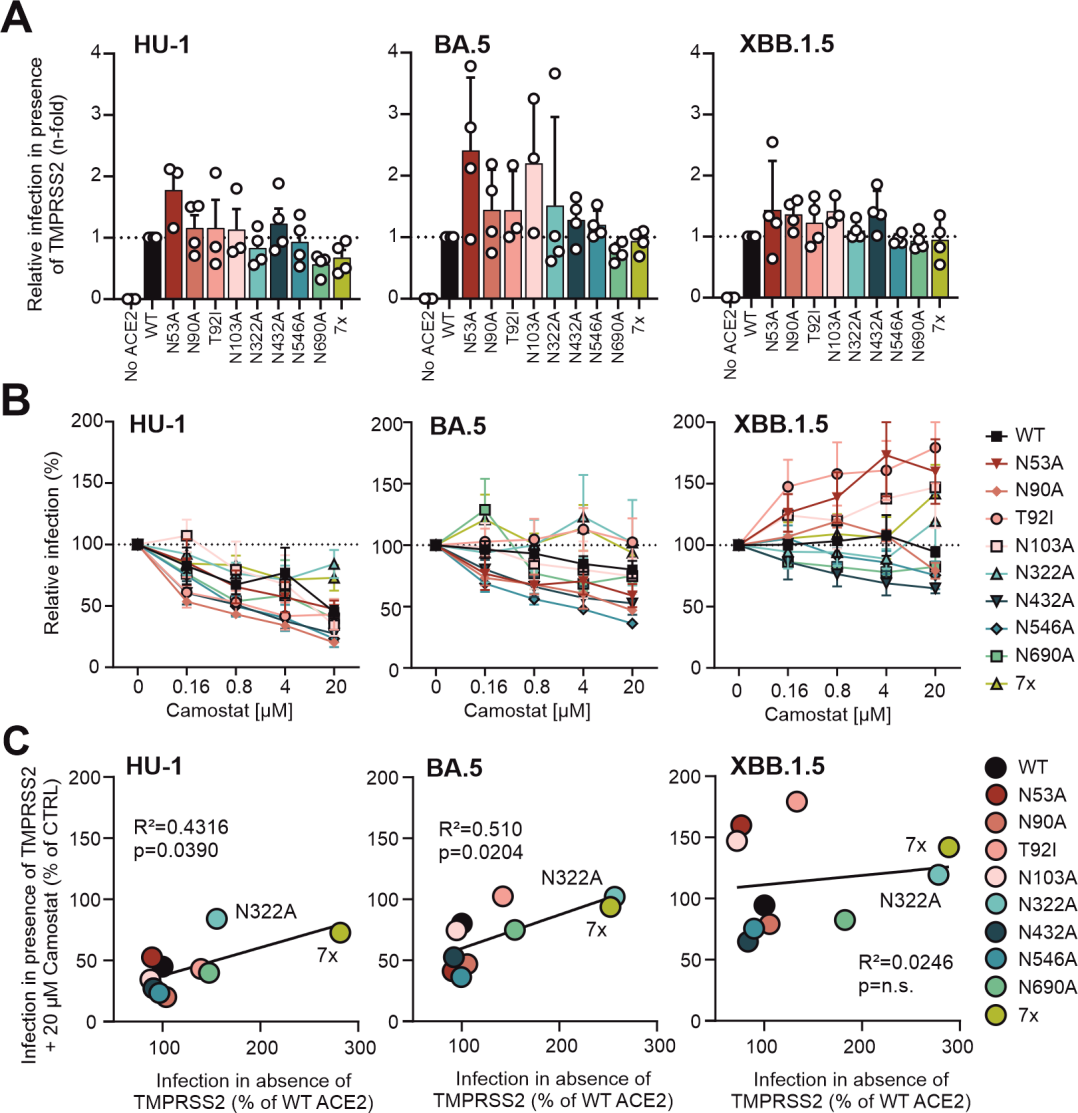
Impact of hACE2 N-glycosylation mutants on SARS-CoV-2 Spike-mediated infection in the presence of TMPRSS2. Cells were cotransfected with the indicated hACE2 constructs and a vector expressing TMPRSS2 and infected with VSVpp carrying SARS-CoV-2 HU-1, BA.5, or XBB.1.5 Spike proteins. (A) Endpoint of SARS-CoV-2 S VSVpp infection after 24 h. Bars represent the mean of four independent experiments (± SEM). (B) Automated quantification of GFP fluorescence of HEK293T cells expressing TMPRSS2 and the indicated forms of hACE2 infected with VSVΔG-GFP pseudotyped with the indicated S variants. Cells were pre-treated (1 h, 37°C) with the indicated concentrations of camostat. Symbols represent the mean of four independent experiments (±SEM). (C) Correlation between HU-1, BA.5, and XBB.1.5 S-mediated VSVpp infection in HEK293T cells expressing ACE2 alone or together with TMPRSS2 in the presence of 20 µM Camostat, relative to the absence of inhibitor (CTRL). Each dot represents the average value derived from four infection experiments (shown in panels A and B). Coefficient of determination (R^2^-values) and two-tailed *P* values are provided.

SARS-CoV-2 can enter cells upon TMPRSS2-mediated S processing at the plasma membrane or by cathepsin-dependent S cleavage and subsequent fusion in endosomes (50). HEK293T cells do not naturally express TMPRSS2 (8, 15). Thus, ACE2-expressing HEK293T cells rely on the endosomal cathepsin-mediated entry pathway (8, 15). To examine the effects of N-glycosylation of hACE2 on S-mediated entry at the cell surface, we cotransfected HEK293T cells with a TMPRSS2 expression construct together with the vectors expressing the various hACE2 proteins. In the presence of TMPRSS2, the lack of specific or all N-glycosylation sites in ACE2 had little if any effect on the entry of VSVpp carrying the HU-1, BA.5, or XBB.1.5 S proteins (Fig. 5A). While infection efficiencies were somewhat variable, the results clearly showed that N322A and lack of all N-linked glycosylation sites in ACE2 only increased infection in the absence (Fig. 4C) but not in the presence of TMPRSS2 (Fig. 5A). The three S proteins showed different susceptibility to inhibition by the TMPRSS2 inhibitor Camostat (51) with HU-1 S being most sensitive, XBB.1.5 S being resistant, and BA.5 S showing an intermediate phenotype (Fig. 5B). In some cases, XBB.1.5 S-mediated entry was even enhanced in the presence of increasing doses of Camostat. It has been previously suggested that blocking TMPRSS2-mediated entry may promote the use of the cathepsin-driven endosomal pathway and potentially increase total infection (28, 52). Notably, changes in ACE2 that increased infection efficiencies in the absence of TMPRSS2 (i.e., N322A and 7×) were associated with reduced sensitivity to inhibition by Camostat in the case of HU-1 and BA.5 S (Fig. 5C). Thus, our results agree with reduced dependency of Omicron variants on TMPRSS2 and suggest that changes in N322A and 7× may shift the site of S-mediated entry from the surface to endosomal compartments.

### Impact of glycans in hACE2 on genuine SARS-CoV-2 and hCoV-NL63 infection

**Fig 6:**
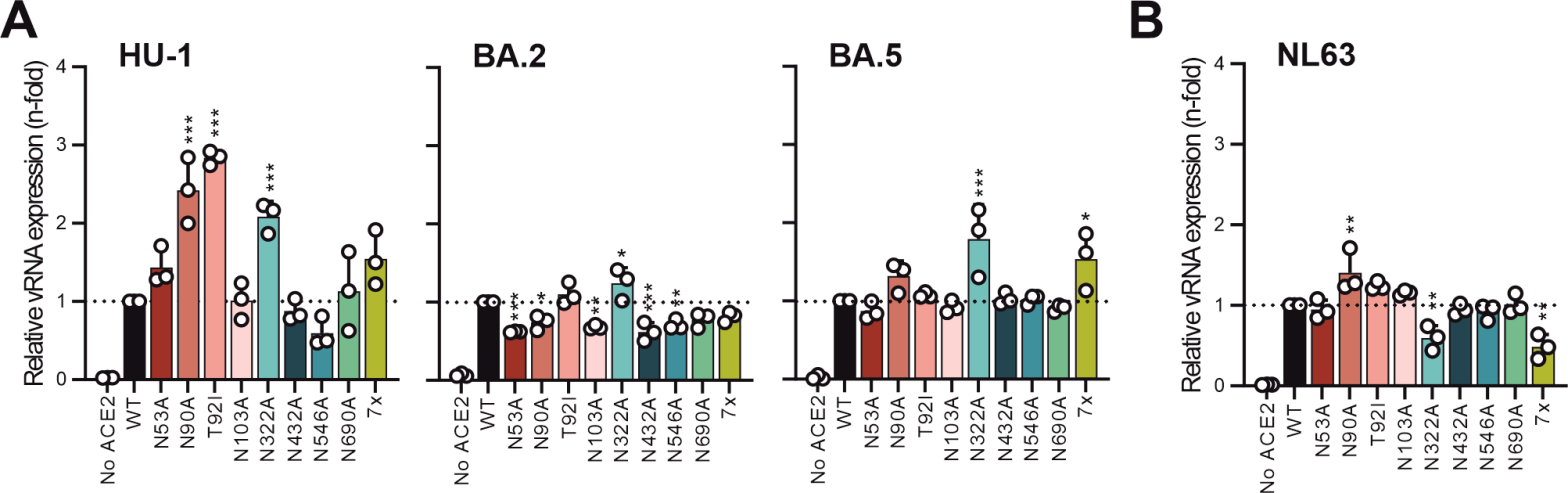
Effect of changes in hACE2 N-linked glycosylation sites on genuine SARS-CoV-2 and hCoV-NL63 infection. (A, B) Transfected and genuinely infected HEK293T cells have been lysed and analyzed via qRT-PCR. Viral Nucleoprotein (N) RNA levels of HEK293T cells infected with a multiplicity of infection (MOI) of 0.01 for indicated SARS-CoV-2 strains (A) and an MOI of 0.05 for hCoV-NL63 infection (B). Cells were harvested at 24 h post-infection, and n-fold SARS-CoV-2 or hCoV-NL63 N RNA levels were normalized to GAPDH. Bars represent the mean of three independent experiments (±SEM). Statistical significance was tested by one-way ANOVA, *P < 0.05; **P < 0.01; ***P < 0.001.

To further determine the impact of hACE2 N-glycosylation on coronavirus infection, we challenged transfected HEK293T cells with genuine SARS-CoV-2 (HU-1, BA.2, and BA.5) and hCoV-NL63 (Fig. 6). Consistent with the results obtained using S-mediated VSVpp infection (Fig. 4), mutation of N322A in hACE2 significantly increased genuine SARS-CoV-2 HU-1, BA.2, and BA.5 infection by the endosomal entry pathway (Fig. 6A). Changes in other hACE2 N-linked glycosylation sites had relatively modest effects on genuine SARS-CoV-2 BA.2 and BA.5 infection (Fig. 6A). By contrast, mutations of N90A and T92I in hACE2 enhanced infection by SARS-CoV-2 HU-1 about threefold. On average, lack of all glycans in hACE2 increased genuine HU-1 and BA.5 infection (Fig. 6A), albeit to a lesser extent than VSVpp infection by the corresponding S proteins (Fig. 4). In comparison to the effects on SARS-CoV-2 infection, mutation of N322A and the complete absence of hACE2 N-glycosylation significantly reduced infection by genuine hCoV-NL63 (Fig. 6B). Thus, N-glycosylation of hACE2 has differential effects on hCoV-NL63 and SARS-CoV-2 infection.

## DISCUSSION

The impact of N-linked glycosylation sites in the SARS-CoV-2 Spike protein on viral infectivity and immune evasion has been well studied (22, 39, 53). In comparison, the role of N-linked glycosylation sites in hACE2 for fusion and entry mediated by the S proteins of human coronaviruses, especially highly relevant SARS-CoV-2 Omicron variants, is poorly understood. Here, we show that the elimination of all N-linked glycosylation sites in hACE2 moderately reduced cell-to-cell fusion mediated by the HU-1, BA.2, BA.5, and XBB.1.5 S proteins. By contrast, lack of glycosylation enhanced S-mediated endosomal infection of VSVpp, especially in the case of Omicron BA.5 and XBB.1.5 subvariants (Fig. 4). Lack of N322 largely recapitulated the effects of the entire lack of glycans. This came as a surprise because it has been previously reported that N-glycosylation is required for efficient SARS-CoV-2 S-mediated entry of VSVpp (28) and that glycans at N90 play the major role in enhanced binding of the S protein to enzymatically deglycosylated hACE2 (25, 30). The previous studies analyzed the S protein of early SARS-CoV-2 strains. Our comparative analyses indicate that both N90- and N322-linked glycans hinder efficient endosomal entry mediated by the S protein of the early SARS-CoV-2 HU-1 strain, while only glycans at N322 inhibit infection by the Omicron BA.5 and XBB.1.5 variants (Fig. 4 and 5). The S proteins of Omicron variants differ by at least 30 amino acid changes from those of early SARS-CoV-2 variants, many of which are located in hACE2 interaction sites. Thus, it is conceivable that individual hACE2 glycosylation sites may have differential relevance for infection by early SARS-CoV-2 strains and later Omicron variants.

It has been reported that N-glycosylation is critical for the proper cell surface expression of hACE2 (28). We also found that mutation of all seven N-linked glycosylation sites reduced cell surface expression of hACE2. However, this effect was modest, and lack of all N-linked glycosylation sites only slightly impaired cell-to-cell fusion mediated by SARS-CoV-2 S proteins (Fig. 3). Despite reduced cell surface expression of the deglycosylated hACE2 receptor, infection mediated by HU-1, BA.5, and XBB.1.5 S proteins was significantly enhanced compared to WT hACE2 in the absence of TMPRSS2 and hardly affected in its presence. In comparison, only modest effects were observed in BA.2 S-mediated endosomal entry, while hCoV-NL63 S-mediated VSVpp and genuine NL63 infection were impaired. Previous studies have established that SARS-CoV-2 S has a substantially higher affinity for hACE2 than NL63 S (54), as well as SARS-CoV-1 S (13, 55), and that hACE2 binding and fusogenicity also increased from BA.2, over BA.5 to XBB.1.5 S (56, 57). In addition, variations in S can affect the threshold levels of ACE2 required for coronavirus infection. For example, it has been reported that mutations like D614G, P681R, or S704L allow SARS-CoV-2 to infect cells at lower levels of ACE2 expression than SARS-CoV-1 (58, 59). Thus, it is tempting to speculate that the impact of hACE2 deglycosylation on entry by ACE2-tropic CoVs depends on the binding affinity and ACE2 threshold requirements of their S proteins. Reduced cell surface expression of the deglycosylated hACE2 receptor will impair infection via S proteins showing relatively low affinity, while lack of steric hindrance and increased flexibility will increase infection by CoVs expressing high-affinity S proteins.

Of the seven glycosylation sites in hACE2, those at N90 and N322 are expected to exert major effects on SARS-CoV-2 and hCoV-NL63 since they may directly interact with the viral S protein (25, 27–30). Initial molecular dynamics simulations suggested that the N322 glycan in hACE2 increases binding to the SARS-CoV-2 RBD (23). Our data show, however, that mutation of N322A in hACE2 strongly enhanced S-mediated VSVpp as well as genuine virus infection, especially in the case of the BA.5 and XBB.1.5 variants analyzed. These findings agree with the accumulating evidence that the N322 glycan sterically hinders the interaction of hACE2 with the SARS-CoV-2 RBD (29, 30), and further indicate that the inhibitory effect of this glycan is particularly pronounced for Omicron variants that continue to dominate around the globe.

Previous studies indicated that the N90-linked glycan in hACE2 forms numerous hydrogen bonds with the SARS-CoV-2 S protein and accounts for a significant part of the interface between hACE2 and the Spike protein (30). Comprehensive mutational analyses confirmed that removal of the N90 glycosylation site increased the affinity of hACE2 for S binding (25). A more recent study confirmed that N90A in hACE2 increases binding affinity as well as SARS-CoV-2 infection more strongly than mutation of N322A (29). In further support for a negative role in CoV-2 infection, hACE2 proteins lacking N90 are highly effective in mediating the entry of human and animal CoVs (60). Unexpectedly, only mutation of N322A but not N90A significantly enhanced infection mediated by the BA.5 and XBB.1.5 S proteins. By contrast, both N90A and N322A increased infection of the early HU-1 strain (Fig. 5). This suggests that Omicron evolved to avoid restriction by N90-glycans, possibly at the cost of increased steric hindrance by N322-glycans. In addition, it has been reported that the N460K mutation that first emerged in Omicron BA.2.75 interacts with the N90 hACE2 glycan, thereby stabilizing the interaction between the S RBD and hACE2 (61). This may explain why lack of N90 is more favorable for early SARS-CoV-2 strains than for the BA.5 and XBB.1.5 strains. We also examined the impact of the T92I variation in hACE2 that is found in the European population with a frequency of 4.5 × 10^−6^ (genomAD browser: rs763395248) (46). Since the T92I variation is very rare, it is not known whether it influenced the susceptibility to SARS-CoV-2 infection or disease outcome during the early phase of the COVID-19 pandemic. Unexpectedly, mutation of T92I but not of N90A significantly increased hACE2-mediated endosomal infection by VSVpp carrying the HU-1, BA.2, or BA.5 S proteins (Fig. 4C). This agrees with previous evidence that the T92I variation increases the affinity of hACE2 for SARS-CoV-2 S binding (21, 29). It also raises the question, however, whether the T92I polymorphism in hACE2 favors SARS-CoV-2 S interaction independently of N-linked glycosylation at this position.

Our study has some limitations. Perhaps most importantly, we overexpressed the hACE2 proteins in HEK293T cells that do not naturally express TMPRSS2 and predominantly support SARS-CoV-2 entry using the endocytic pathway via cathepsins (8, 15). High expression levels and the presence of TMPRSS2 (Fig. 5) may reduce the impact of N-linked glycosylation sites in hACE2 on its ability to serve as entry cofactors for SARS-CoV-2 or hCoV-NL63. In addition, the types of glycans vary between different cell types (62). Thus, it will be important to challenge the findings in human lung cells. Furthermore, the results obtained using genuine SARS-CoV-2 strains and VSVpp containing the corresponding S proteins were not fully consistent. For example, mutation of N690A in hACE2 generally increased S-mediated VSVpp infection about 1.5-fold (Fig. 4) but had no significant effect on genuine SARS-CoV-2 and hCoV-NL63 infection (Fig. 6). N690 is located in the so-called neck domain that mediates hACE2 dimerization (16) and may hence indirectly affect S-mediated VSVpp infection. Altogether, it seems that S-containing VSVpp is more sensitive to changes in N-linked glycosylation sites compared to genuine CoVs. Further studies are required to address this and to clarify whether differences in S protein content, absence of the CoV E (Envelope) and M (Membrane) proteins in VSVpp, virion morphology, and/or differential susceptibility to innate defense mechanisms may contribute to these differences.

In conclusion, we show that lack of N-linked glycosylation sites in hACE2 enhances SARS-CoV-2 infection and that N322 plays the key role in suppressing endosomal infection of Omicron variants, while N90 seems to mainly affect early viral strains. This knowledge may help to generate mutant hACE2 proteins that efficiently neutralize currently dominant SARS-CoV-2 variants.

## MATERIALS AND METHODS

### Cell culture

All cells were cultured at 37°C and 5% CO_2_ in a humified atmosphere. HEK293T (human embryonic kidney) cells (ATCC: #CRL3216) and HeLa (Henrietta Lacks) cells (ATCC: #CCL-2) were maintained in Dulbecco’s Modified Eagle Medium (DMEM, Gibco, Cat#41965039) supplemented with 10% (vol/vol) heat-inactivated fetal bovine serum (FBS, Gibco, Cat#10270106), 2 mM L-glutamine (PANBiotech, Cat#P04-80100), 100 µg/mL streptomycin (PANBiotech, Cat#P06-07100) and 100 U/mL penicillin (PANBiotech, Cat#P06-07100). Mouse I1-Hybridoma cells (ATCC: #CRL2700) were cultured in Roswell Park Memorial Institute (RPMI) 1640 medium (Gibco, Cat#21875034) supplemented with 10% (vol/vol) heat-inactivated fetal bovine serum (Gibco), 2 mM L-glutamine (PANBiotech), 100 µg/mL streptomycin (PANBiotech) and 100 U/mL penicillin (PANBiotech). Calu-3 (human epithelial lung adenocarcinoma, kindly provided by Prof. Manfred Frick [Ulm University]) cells were cultured in Minimum Essential Medium Eagle (MEM, Sigma, Cat#M4655) supplemented with 10% (upon and after viral infection) or 20% (during all other times) heat-inactivated fetal bovine serum (FBS, Gibco, Cat#10270106), 100 units/mL penicillin, 100 µg/mL streptomycin (ThermoFisher, Cat#15140122), 1 mM sodium pyruvate (PANBiotech, Cat#P04-8010), and 1× non-essential amino acids (Sigma, Cat#M7145). Vero E6 cells (Cercopithecus aethiops derived epithelial kidney, ATCC) and TMPRSS2-expressing Vero E6 cells (kindly provided by the National Institute for Biological Standards and Control (NIBSC, No. 100978) were grown in Dulbecco’s modified Eagle’s medium (DMEM, Gibco, Cat#41965039) supplemented with 2.5% (upon and after viral infection) or 10% (during all other times) heat-inactivated FBS (Gibco, Cat#10270106), 100 units/mL penicillin, 100 µg/mL streptomycin (ThermoFisher, Cat#15140122), 2 mM L-glutamine (Gibco, Cat#25030081), 1 mM sodium pyruvate (PANBiotech, Cat# P04-8010), 1× non-essential amino acids (Sigma, Cat#M7145), and 1 mg/mL Geneticin (Gibco, Cat#10131–019) (for TMPRSS2-expressing Vero E6 cells).

### SARS-CoV-2 stocks

The SARS-CoV-2 variant B.1.1.529, BA.5 (Omicron BA.5), was kindly provided by Prof. Dr. Florian Schmidt and Dr. Bianca Schulte (University of Bonn). The BetaCoV/Netherlands/01/Nl/2020 (NL-02–2020) lineage was obtained from the European Virus Archive. The SARS-COV-2 hCoV-19/USA/CO-CDPHE-2102544747/2021, lineage B.1.1.529, BA.2 (Omicron BA.2) was obtained from the BEI database (Cat, #NR-56520). SARS-CoV-2 strains were propagated on Calu-3 or Vero E6 (NL-02–2020), or Vero E6-TMPRSS2 (for Omicron variants) cells. To this end, 70%–90% confluent cells in 75 cm² cell culture flasks were inoculated with the SARS-CoV-2 isolate (multiplicity of infection [MOI] of 0.03–0.1) in a 3.5 mL serum-free medium. The cells were incubated for 2 h at 37°C before adding 20 mL medium containing 15 mM HEPES (Carl Roth, Cat#6763.1). Virus stocks were harvested as soon as strong cytopathic effect (CPE) became apparent. The virus stocks were centrifuged for 5 min at 1,000 *g* to remove cellular debris, aliquoted, and stored at −80°C until further use. All procedures involving genuine SARS-CoV-2 were performed in the BSL3 facilities of Ulm University in accordance with institutional biosafety committee guidelines.

### Expression constructs

hACE2 encoding the hACE2 protein (NCBI reference Sequence LC698008.1) was synthesized by Twist Bioscience, PCR amplified, and cloned into a pCG expression construct using the restriction enzymes XbaI and MluI. pCG_hACE2 N-linked glycosylation mutant plasmids were generated using the Q5 Site-directed Mutagenesis Kit (NEB #E0554). The hACE2 combined mutant was synthesized by Twist Bioscience, PCR amplified, and subcloned into a pCG expression construct using the restriction enzymes XbaI and MluI. pCG expression plasmids encoding for the spike protein of SARS-CoV-2 isolate Wuhan-HU-1 (NCBI reference Sequence YP_009724390.1), pCG1_SARS-2-SΔ18 (BA.2), and pCG1_SARS-2-SΔ18 (BA.4/5) were kindly provided by Stefan Pöhlmann (DPZ Göttingen, Germany). The Spike sequence of all constructs was PCR amplified and subcloned into a pCG-IRESeGFP expression construct by Gibson Assembly repairing the C-terminal deletion and introducing the V5 epitope tag. hCoV-NL63 Spike was synthesized by Twist Bioscience, PCR amplified, and subcloned into a pCG-BFP expression construct using the restriction enzymes XbaI and MluI. All constructs were verified by sequence analysis using a commercial sequencing service (Eurofins Genomics).

### Pseudoparticle production

To produce pseudotyped VSVΔG(GFP) particles, 1 million HEK293T cells were transfected with 1 µg SARS-CoV-2 and hCoV-NL63 Spike-expressing vectors using polyethyleneimine (PEI 1 mg/mL in H_2_O). After 22 h of transfection, the cells were infected with VSVΔG(GFP)*VSV-G at an MOI of 3. Pseudotyped VSVΔG-GFP particles were harvested 16 h post-infection. The remaining cell debris was removed by centrifugation (500 × *g* for 5 min). Residual particles carrying VSV-G were blocked by adding 10% (vol/vol) of I1-Hybridoma supernatant (I1, mouse hybridoma supernatant from CRL-2700; ATCC) to the cell culture supernatant.

### Transfections

In all, 500,000 or 250,000 HEK293T cells were transfected with either 0.5 µg or 0.25 µg plasmid DNA for native infection or VSV-ΔG S pps-based infection, respectively, using polyethylenimine (PEI, 1 mg/mL in H_2_O, Sigma-Aldrich) according to the manufacturer’s protocol.

### Genuine SARS-CoV-2 infection

In all, 500,000 HEK293T cells were transfected with 0.5 µg pCG_hACE2 constructs, and 24 h later, medium was changed to DMEM with 2.5% FCS and cells infected with indicated SARS-CoV-2 strains at an MOI of 0.05, or hCoV-NL63 at an MOI of 0.01. After 3 h of infection, the input virus was removed, and cells were washed twice with PBS and supplemented with fresh medium. For qRT-PCR analysis, cells were harvested 24 h post-infection.

### Camostat treatment

In all, 250,000 HEK293T cells were transfected with 0.125 µg TMPRSS2 DNA (Addgene 53887, kindly provided by Roger Reeves, Johns Hopkins University, Baltimore, USA) and 0.125 µg of respective pCG_hACE2 constructs. 24 h post-transfection, cells were treated with four serial dilutions (20 µM, 4 µM, 0.8 µM, and 0.16 µM) of the TMPRSS2 inhibitor Camostat (Camostat mesylate, Sigma Aldrich, Cat#SML0057), or were left untreated. 1 h post-treatment, HEK293T cells were infected with HU-1 S, BA.5 S, and XBB.1.5 S VSVpp which were produced as previously described. 24 h post-infection, SARS-CoV-2-infected GFP-positive cells were quantified using the Cytation 3 plate reader microscope.

### Whole-cell lysates

To prepare whole-cell lysates, cells were collected and washed in phosphate-buffered saline (PBS), pelleted, and lysed in transmembrane lysis buffer (150 mM NaCl, 50 mM HEPES, 5 mM EDTA, 1% Triton X-100), containing protease inhibitor (1:500, Roche, Cat#04693132001). After 5 min of incubation on ice, samples were cleared by centrifugation (4°C, 20 min, 20,817 × *g*). Supernatants were transferred to new tubes, mixed with 4× Protein Sample Loading Buffer (LI-COR, Cat#928–40004) containing 10% β-Mercaptoethanol, and denatured at 95°C for 10 min.

### SDS-PAGE and immunoblotting

Whole-cell lysates were loaded on a NuPAGE 4-12% Bis-Tris Gel (Invitrogen) and run for 90 min at 120 V 1  ×  MES SDS running buffer (Invitrogen, Cat#NP0002). Subsequently, the gel was blotted at constant 30 V for 30 min onto methanol-activated PVDF-Membrane (Immobilon-FL, Merck, Cat#IPFL00010) using 1× semi-dry blot transfer buffer (Alfa Aesar, Cat#J63664.K3). Afterward, the membrane was blocked using 1% (wt/vol) casein (Thermo Scientific, Cat#37528) for 1 h at room temperature (RT), then incubated with primary antibodies, diluted in PBS-T containing 0.05% (wt/vol) casein (Thermo Scientific) overnight at 4°C. The membrane was extensively washed with PBS-T (PBS containing 0.2% [vol/vol] TWEEN-20, Sigma-Aldrich). The respective secondary antibodies (IRDye Secondary Antibodies, LI-COR) were incubated with the membrane for 30 min at RT, followed again by extensive washing with PBS-T for 30 min. The blot was developed on an Odyssey 9120 (LI-COR) infrared imager, and band intensities were quantified using LI-COR Image Studio version 5. The following antibodies were used: anti-ACE2 (1:1,000, #GTX01160, GeneTex), anti-ACE2 (1:1,000, #MAB9332, R&D Systems), anti-SARS-CoV-2 N (1:1,000, #40143-R004A, Sino Biological and 1:1000, #40143-MM05, Sino Biological), anti-NL63 N (1:500, #40641-T62, Sino Biological), anti-GAPDH (1:2,000, #W17079A, BioLegend), and anti-β-actin (1:2,000, AC-15, Invitrogen) as primary antibodies and IRDye 800CW Goat anti-Mouse (#926-32210), IRDye 800CW Goat anti-Rabbit (#926-32211), IRDye 800CW Goat anti-Rat (#926-32219), IRDye 680RD Goat anti-Rabbit (#925-68071), and IRDye 680RD Goat anti-Mouse (#926-68070) as secondary antibodies.

### qRT-PCR

To determine virus infection, cellular RNA was isolated using the Viral RNA Mini Kit (Qiagen, Cat#52904) according to the manufacturer’s instructions. qRT-PCR was performed according to the manufacturer’s instructions as well using TaqMan Fast Virus 1-Step Master Mix (Thermo Fisher, Cat#4444436) and a OneStepPlus Real-Time PCR System (96-well format, fast mode). Primers and NL63 N Probe were purchased from Biomers and dissolved in RNAse-free water. Primers and GAPDH Probe for internal control were ordered from ThermoFisher Scientific (#4310884E). Synthetic SARS-CoV-2 RNA (Twist Bioscience) was used as a quantitative standard to obtain viral copy numbers. All reactions were run in duplicates. Forward primer SARS-CoV-2 N HKU-NF: 5′-TAA TCA GAC AAG GAA CTG ATT A-3′; Reverse primer HKU-NR: 5′-CGA AGG TGT GAC TTC CAT G-3′; Probe HKU-N: 5′-FAM-GCA AAT TGT GCA ATT TGC GG-3′TAMRA. Forward primer NL63 N 63NF2: 5′-AAA CCT CGT TGG AAG CGT GT-3′; Reverse primer 63NR1: 5′-CTG TGG AAA ACC TTT GGC ATC-3′; Probe 63 NP: 5′-FAM-ATG TTA TTC AGT GCT TTG GTC CTC GTG AT-TAMRA-3′.

### Flow cytometry

To determine the amount of surficially expressed hACE2 via flow cytometry, 500,000 HeLa cells were transfected with 0.5 µg hACE2 constructs, and 24 h later harvested, washed in PBS, and fixed and permeabilized at the same time using the BD Cytofix/Cytoperm Fixation/Permeabilization Kit (Thermo Fisher Scientific, #BDB554714) according to the manufacturer’s instructions. Cells were stained using Fixable Viability Dye eFluor 450 (eBioscience, #65-0863-14), anti-ACE2 AF647 (Abcam, #ab283658), and anti-IgG Isotype control AF647 (Abcam, #ab199093) antibodies, and flow cytometric measurements were performed using a BD FACS Canto II flow cytometer. The data were analyzed with FlowJo Version 9.

### Confocal microscopy

In all, 500,000 HeLa cells were plated in 24-well tissue culture dishes on 13 mm round borosilicate coverslips (VWR, Cat#6310150), pre-coated with poly-L-lysine. After 24 h, the cells were transfected with 0.5 µg expression constructs for hACE2 protein using polyethyleneimine (PEI 1 mg/mL in H_2_O). After 24 h of transfection, cells were washed with cold PBS and fixed in 4% paraformaldehyde solution (PFA, Santa Cruz, Cat#281692) for 20 min at RT, permeabilized and blocked in PBS containing 0.5% Triton X-100 (Sigma-Aldrich, Cat#T9284) and 5% FCS (Gibco) for 1 h at RT. Thereafter, samples were washed with PBS and incubated for 2 h at 4°C with primary antibody (anti-ACE2 [1:100, #GTX01160, GeneTex]) diluted in PBS. The samples were washed with PBS/0.1% Tween 20 and incubated in the dark for 2 h at 4°C with the secondary antibody (Alexa Fluor-647-conjugated anti-rabbit antibody, 1:1,000, Thermo Fisher Scientific, Cat#A-21244) and 500 ng/mL DAPI (Sigma Aldrich, Cat#D9542). After washing with PBS-T and water, coverslips were mounted on microscopy slides in Mowiol mounting medium (Mowiol 4%–88 10% [wt/vol, Carl Roth, Cat#0713.1], glycerol 25% [wt/vol, Sigma-Aldrich, Cat#G5516], H_2_O 25% [vol/vol], 0.2 M Tris HCl pH 8.5 50% [vol/vol, AppliChem GmbH, VWR, Cat#A3452], DABCO 2.5% [wt/vol, Carl Roth, Cat# 0718.1]). Images were acquired using a Zeiss LSM800 confocal laser scanning microscope with ZEN imaging software (ZEN 2.3 SP1, Zeiss).

### Quantification of syncytia formation

To detect the formation of syncytia, 700,000 HEK293T cells were co-transfected with 0.5 µg ACE-2 and 0.5 µg Spike expressing vectors using polyethyleneimine (PEI 1 mg/mL in H_2_O). After 24 h of transfection, fluorescence microscopy images were acquired, and GFP+ cells were counted automatically using the Cytation 3 microplate reader (BioTek Instruments). The GFP area was quantified using ImageJ (ImageJ 2.0).

### Statistical analysis

Statistical analyses were performed using GraphPad PRISM 10.0.2 (GraphPad Software). *P*-values were determined using a two-tailed Student’s t-test with Welch’s correction or Brown-Forsythe/one-way ANOVA with multiple comparisons. Unless otherwise stated, data are shown as the mean of at least three independent experiments ± SEM. Significant differences are indicated as *P < 0.05; **P < 0.01; ***P < 0.001; ****P < 0.0001.

## SUPPLEMENTAL MATERIAL

Supplemental figures jvi.02202-24-s0001.pdf Figures S1 to S4.

ASM does not own the copyrights to Supplemental Material that may be linked to, or accessed through, an article. The authors have granted ASM a non-exclusive, world-wide license to publish the Supplemental Material files. Please contact the corresponding author directly for reuse.
