# Supplementary material for: Role of N-linked glycosylation sites in human ACE2 in SARS-CoV-2 and hCoV-NL63 infection
Source: J Virol. 2025 Mar 28;99(5):e02202-24. doi: 10.1128/jvi.02202-24 (PMC12090729; doi:10.1128/jvi.02202-24)
Supplement: Supplemental figures — Figures S1 to S4. [file jvi.02202-24-s0001.pdf]

Role of N-linked glycosylation sites in human ACE2 in SARS-CoV-2 and hCoV-NL63 infection

Sabrina Noettger, Fabian Zech, Rayhane Nchioua, Chiara Pastorio, Christoph Jung, Timo Jacob, Steffen Stenger and Frank Kirchhoff

Four supplemental figures

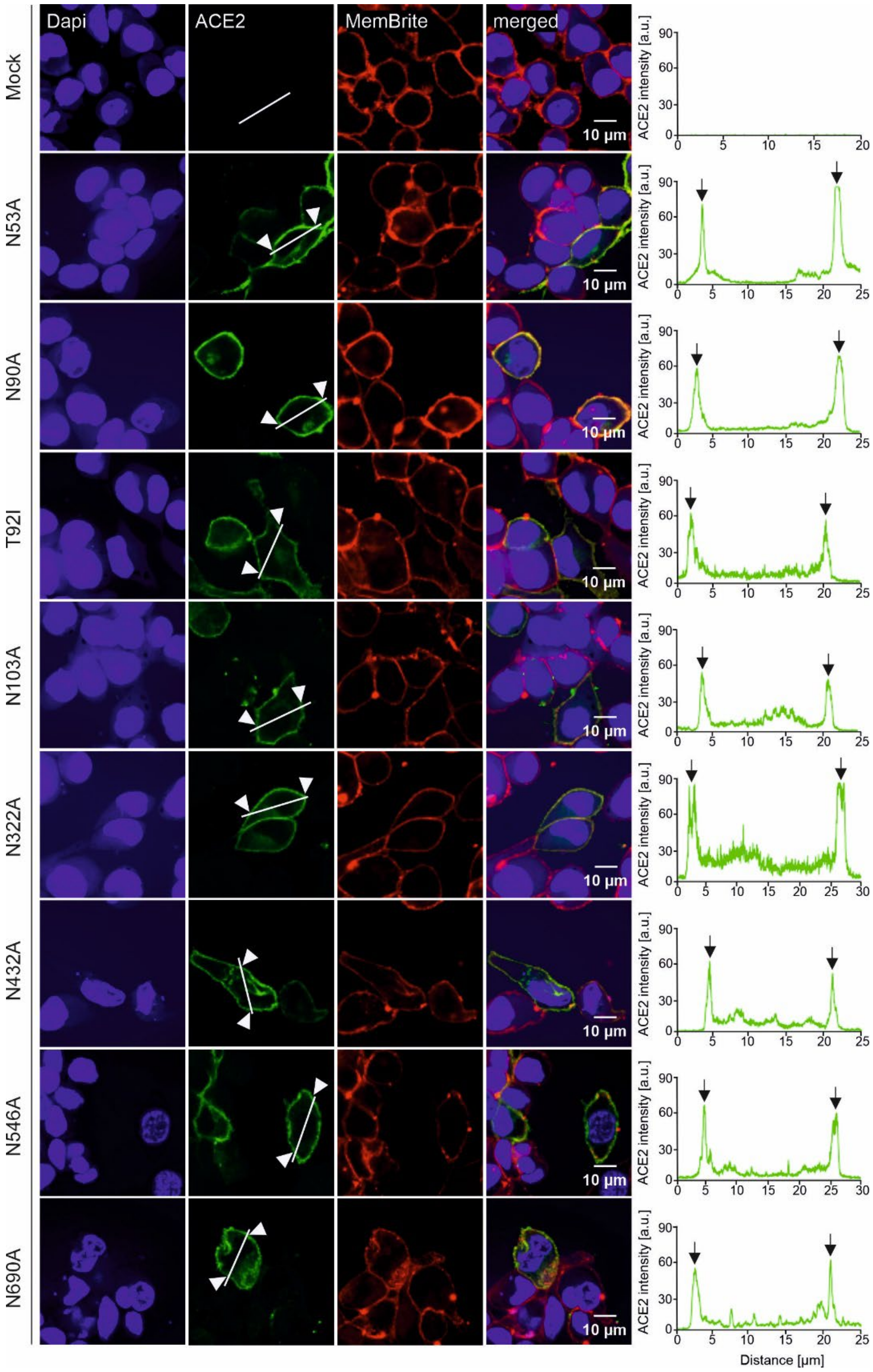

Figure S1. Localization of hACE2 N-glycosylation mutants. Immunofluorescence staining and intensity plots of HeLa cells expressing single N-glycosylation mutants or empty vector control (Mock). Cell nuclei are stained in blue (DAPI), the cell membrane in red (MemBrite®), and ACE2 in green. Intensity plots show the intensity and localization of the ACE2 fluorescent signal throughout one cell. The x-axis represents the distance (in µm) along the cell, the y-axis denotes the intensity of the ACE2 fluorescence signal. The white arrows in the images and black arrows in the intensity blots indicate the position of the cellular membrane.

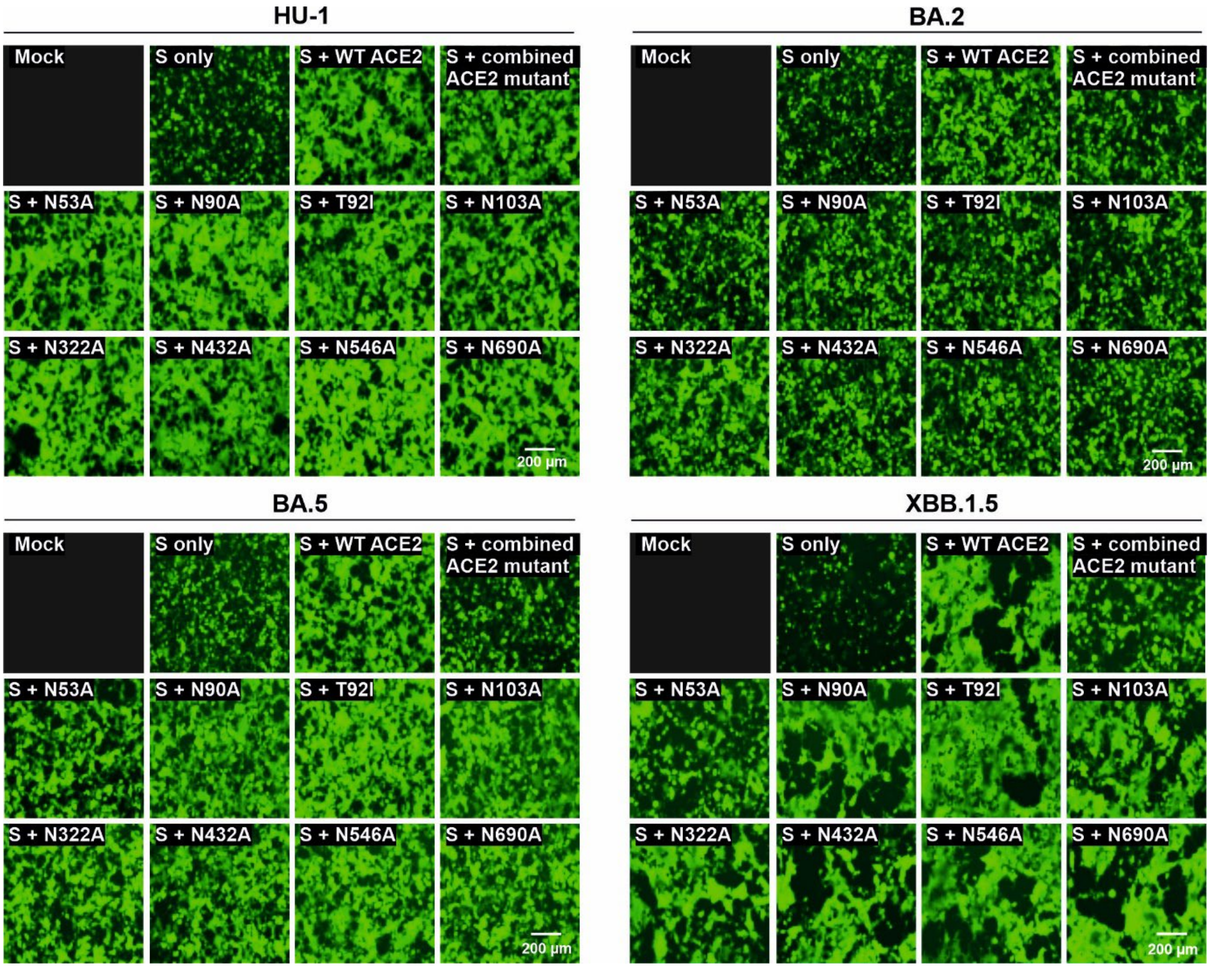

Figure S2. SARS-CoV-2 XBB.1.5 S forms large syncytia with HEK293T cells expressing various ACE2 constructs which are mutated in their N-glycosylation sites respectively. Representative visual depiction of ACE2-S cell-to-cell fusion events during syncytia formation. Cells are expressing indicated ACE2 mutants and HU-1, BA.2, BA.5, and XBB.1.5 S protein respectively. SARS-CoV-2 S proteins harbor an eGFP reporter gene. Pictures were taken with a Cytation 3 plate reader microscope. Scale bar 200 µm.

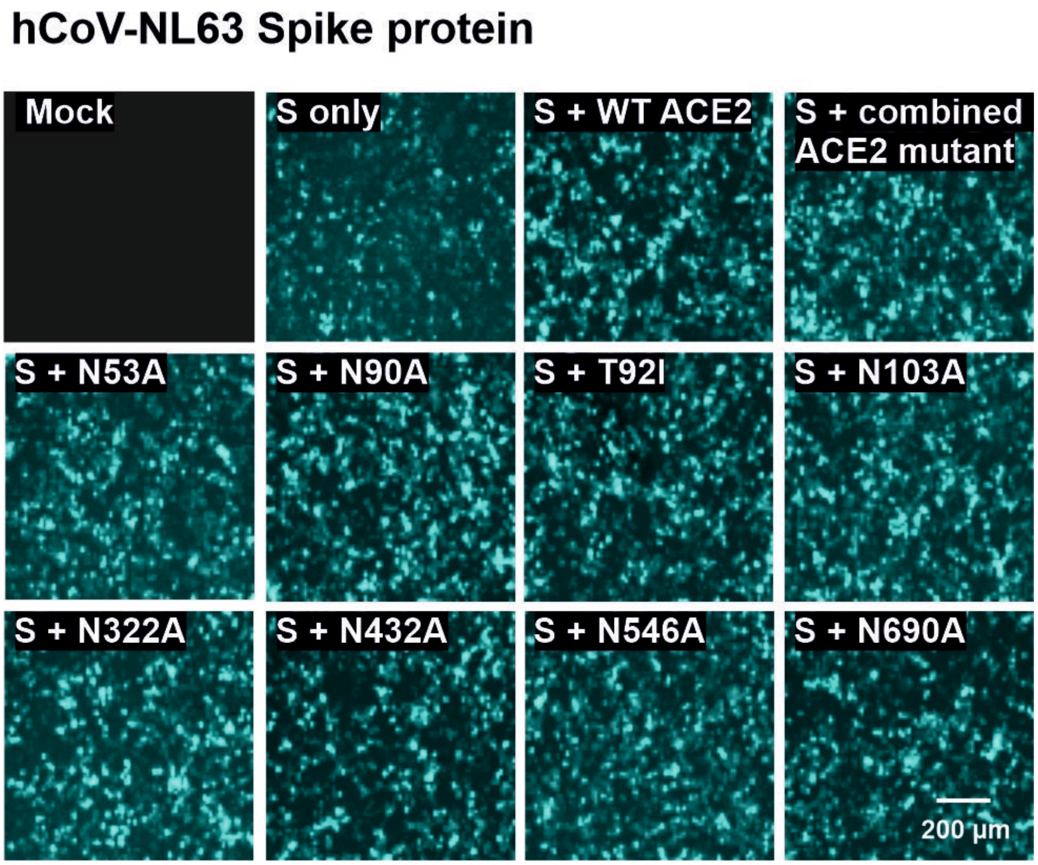

Figure S3. hCoV-NL63 S does not form syncytia with HEK293T cells expressing various ACE2 constructs which are mutated in their N-glycosylation sites respectively. Representative visual depiction of ACE2-S cell-to-cell fusion events during syncytia formation. Cells are expressing indicated ACE2 mutants and hCoV-NL63 S protein. hCoV-NL63 S protein harbors an BFP reporter gene. Pictures were taken with a Cytation 3 plate reader microscope. Scale bar 200 µm.

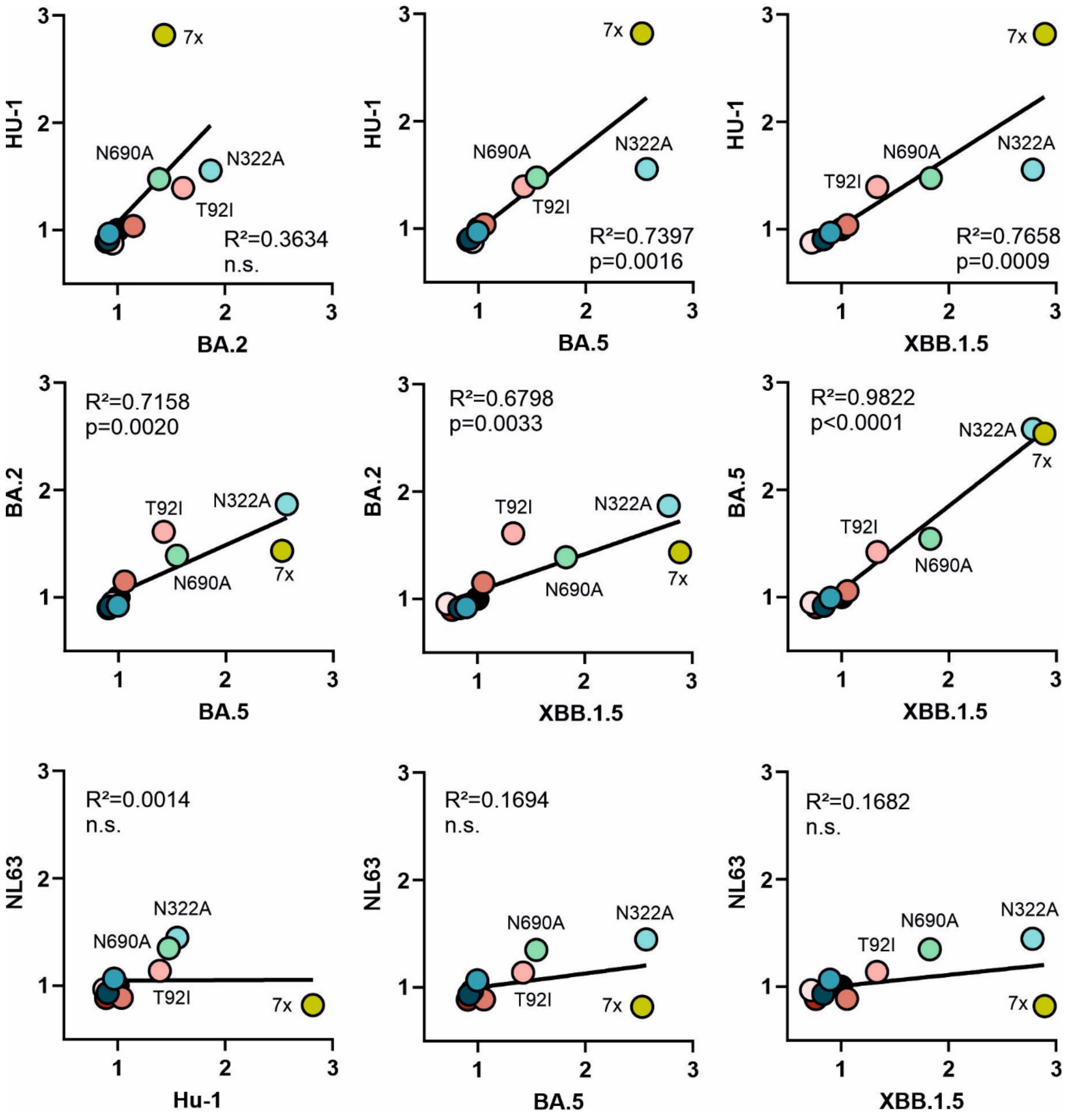

Figure S4. Changes in ACE2 N-linked glycosylation sites have similar effects on entry by the four different SARS-CoV-2 S proteins. Correlation analysis of SARS-CoV-2 and hCoV-NL63 pseudotype infection events of parental and indicated mutant ACE2 constructs. The coefficient of determination (R^2 values) and two-tailed p values are provided.
